# Psychometric properties of satisfaction with the childbirth education class questionnaire for Iranian population

**DOI:** 10.1186/s12884-020-03349-1

**Published:** 2020-11-05

**Authors:** Robab Hassanzadeh, Mohammad Asghari Jafarabadi, Fatemeh Abbas-Alizadeh, Shahla Meedya, Sakineh Mohammad-Alizadeh-Charandabi, Mojgan Mirghafourvand

**Affiliations:** 1 Students’ Research Committee, Tabriz University of Medical sciences, Tabriz, Iran; 2 Road Traffic Injury Research Center, Faculty of Health, Tabriz University of Medical Sciences, Tabriz, Iran; 3 Reproductive Health Research Center, Tabriz University of Medical Sciences, Tabriz, Iran; 4 Member of South Asia Infant Feeding Research Network (SAIFRN), School of Nursing, Faculty of Science, Medicine and Health, University of Wollongong, Wollongong, Australia; 5 Midwifery Department, Nursing and Midwifery Faculty, Tabriz University of Medical Sciences, Tabriz, Iran; 6 Social determinants of Health Research Center, Tabriz University of Medical sciences, Tabriz, Iran

**Keywords:** Satisfaction, Childbirth education, Validity, Reliability, Psychometric, Iran

## Abstract

**Background:**

Childbirth preparation classes can reduce pregnant women’s anxiety and fear for their childbirth. However, to evaluate women’s feedback and their satisfaction with these classes, there is a need for a standard instrument that is suitable for Iranian context. This study is aimed to translate and conduct a psychometric analysis of the Satisfaction with the Childbirth Education Class Questionnaire (SCECQ) for Iranian population.

**Methods:**

The questionnaire was translated from English into Persian through the forward-backward translation method. The cluster sampling method was employed to select 205 pregnant women with gestational age of 35–37 weeks from all health complexes of Tabriz, Iran. The face, content, and construct validity of the research instrument were assessed through exploratory and confirmatory factor analyses. Internal consistency and test-retest reliability were measured to evaluate the overall reliability of the questionnaire.

**Results:**

The impact scores of all items were above 1.5. The content validity index (CVI) and content validity ratio (CVR) of the questionnaire were 0.88 and 0.94, respectively. The convergent construct validity of the whole questionnaire and those of its three subscales were confirmed through the exploratory factor analysis (EFA). The factor loadings of no items were below 0.3, and the X^2^/df ratio was smaller than 5. The overall model validity was confirmed by having the Root Mean Square Error of Approximation (RMSEA) smaller than 0.08. Cronbach’s alpha and intraclass correlation coefficient (ICC) were 0.93 and 0.96, respectively, indicating the acceptable reliability of the questionnaire.

**Conclusion:**

The Persian version of this questionnaire, entitled SCECQ is a valid and reliable instrument for measuring Iranian women’s satisfaction with childbirth education classes.

**Supplementary Information:**

The online version contains supplementary material available at 10.1186/s12884-020-03349-1.

## Background

Due to the worldwide increase in cesarean section (CS) rate, international policies are aimed at encouraging vaginal birth [1]. World Health Organization (WHO) has reported ideal rate of 10 to 15% for CS [2]. However, according to a systematic review, the estimated rate of CS in Iran is very high (48%) and three major factors influence the incidence of CS: a) social and demographic factors such as maternal education and grand multiparty, b) obstetric and medical factors such as having a previous CS, and c) non-obstetric and medical factors such as fear of natural vaginal birth [3]. Other studies also reported that women’s fear of vaginal birth and labor pain as the most important reasons for preferring CS without any medical reasons [1, 4, 5].

In 1960s, Lamaze introduced prenatal maternal education and active participation of mothers in the childbirth process to emphasize natural birth without unnecessary interventions [6, 7]. The results of empirical studies, demonstrated that women who participated in childbirth preparation classes managed to adapt better to labor pain, used fewer labor medicines, and had fewer instrumental deliveries [8–10]. Today, women tend to manage their own labor, control their delivery, use non-pharmacological pain management techniques, and experience a good delivery by sharing this special experience with their family [11]. Lee and Holroyd’s findings showed that pregnant women attending childbirth preparation classes could better manage their pregnancy, childbirth, and postpartum period than those who merely received routine care [12].

In Iran, prenatal care services used to be limited to regular antenatal check and routine tests which was not sufficient to enhance women’s knowledge and reduce their fear of childbirth. Evidence demonstrated that not knowing what to expect during childbirth can led to maternal anxiety and increase rate of medical interventions [13]. Since 2008, the Iranian Ministry of Health and Medical Education introduced a national physiological delivery preparation classes for first time mothers to enhance the quality of maternity care. The classes are free and held in eight sessions from week 20 to week 37 of pregnancy. Based on women’s gestational age, the following topics are covered in the classes: anatomy of reproductive system, physiological adaptations during pregnancy, fetal development, prenatal care and nutrition, physical and mental health, pregnancy risk factors, benefits of natural childbirth, pain relief techniques, postpartum examinations and risk factors, and infant care. Women also learn about various skills such as stretching exercises, posture correction exercises, relaxation methods, massages, and breathing techniques [14]. Although similar childbirth classes have been offered to women in Turkey [15], Australia [16] and Italy [17] for many years, few studies have evaluated mothers’ satisfaction with these classes [12, 17]. Mothers’ satisfaction is an important indicator of antenatal education effectiveness [18], but there is no Iranian study that have explored women’s feedback and satisfaction about their childbirth classes.

One of the reasons for the absence of a broad evaluation on childbirth classes and women’s satisfaction in Iran is the lack of having a standardized tool. In collaboration with Hong Kong and Australia, Lee et al. developed a self-reported Satisfaction with the Childbirth Education Class Questionnaire (SCECQ) to evaluate Chinese mothers’ satisfaction with prenatal education classes. The SCECQ has 25 items which is consisted of three subscales for structure, process, and outcome of the childbirth preparation classes [12]. We didn’t find any other instrument that could comprehensively evaluate women’s satisfaction with their childbirth preparation classes. Ricchi et al. developed an instrument for postpartum period education without evaluating the structure and process of the classes (17).

## Methods

### Aim

This study aimed to translate and assess the psychometric properties of SCECQ**.**

### Sample size

A total number of 5 samples per item were selected as the participants (125 samples for the 25-item questionnaire); however, considering the design effect of the cluster sampling method and a 10% loss to follow-up rate, 205 individuals were selected as the sample.

### Instrument

SCECQ was utilized to collect data on mothers’ satisfaction with childbirth classes. This questionnaire was developed by Lee et al. in Chinese language and was translated in English for the purpose of publications. However, there is no information about the Chinese-English translation process they used [12]. In this 25-item instrument, all items are scored on a five-point Likert scale ranging from “not at all satisfied” (score 1) to “very satisfied” (score 5). The thematic domains of the questionnaire include the class structure (questions 1–5), the class process (questions 6–21), and the class outcome (questions 22–25). The questionnaire demonstrated good content validity with an index of 0.88. The Cronbach alpha for the whole scale was 0.89 and for the structure, process and outcome subscales were 0.76, 0.88 and 0.72 respectively [12]. The present study is proposing an Iranian version of the instrument. This questionnaire has been used in this study after obtaining license. The English and Persian versions of the questionnaire are available as supplementary files.

### Translation process

The translation process was conducted systematically by applying the forward and back-translation method [19], the original version of the instrument was first translated from English into Persian language by a translator, who was a native speaker for both languages. After reviewing the first version of the questionnaire, the Persian version of the instrument was translated into English by other two professional translators who were not involved in the previous stage. The final questionnaire was prepared after review of two independent individuals who were familiar with medical terms and mastered both languages.

### Data collection

The research protocol of this study was published in 2019 [20]. The study was conducted between July 2019 to March 2020 during a 9-months period (one month for questionnaire translation, one month for content and face validity assessment, five months for sampling, and two months for data analysis and manuscript writing). Seven out of 20 healthcare complexes in Tabriz were selected through simple random method. Each complex covered four to five healthcare centers, and sampling was conducted in a total of 29 centers. The required sample size for each complexes was determined using the proportional sampling method. The participants were randomly selected based on quotas determined for each center. The inclusion criteria for the study were being 35 to 37 week pregnant, living in Tabriz city with no age or language restrictions. Women who had not attended any sessions were excluded from the study. After the inclusion of the eligible women, the study objectives and methods were fully explained to them and socio-demographic questionnaire and SCECQ were completed through interviews. The socio-demographic questionnaire was developed by researchers and included the following items: mother’s age, her educational degree, and her job, her spouse’s educational qualifications and job, her household income status, and having a wanted or unwanted pregnancy. The validity of this questionnaire was confirmed through the measurement of its content validity.

### Face and content validity

To assess the face validity of the questionnaire, 20 randomly selected pregnant women were asked to rate the difficulty, relevance, and ambiguity of all questions. The responses were scored on a four-point Likert scale ranging from 1 (completely difficult/irrelevant/ambiguous) to 4 (completely simple/relevant/unambiguous). The respective impact score for each item was calculated through multiplying the mean score of each item (importance) to the number of responses (frequency). An item was considered acceptable, if the impact score for that item was more than 1.5 [19].

Content validity was assessed through qualitative and quantitative methods. In the qualitative phase, 10 midwifery and reproductive health specialists were asked to review the translated questionnaire and provide feedback on the correct grammar, vocabulary, and phrases in each sentence. CVI and CVR were used in the quantitative phase. CVI values were calculated through determining the simplicity, relevance, and unambiguity of the items and scoring them on a four-point Likert scale. A CVI value of higher than 0.79 was considered acceptable. To determine CVR scores, the experts were asked to comment on the necessity of each item by using a four-point scale. Based on the Lawshe Table, the minimum acceptable CVR value was determined as 0.62.

### Construct validity

A scale-based EFA and a CFA were performed to assess the construct validity of the questionnaire. Factor analyses were also performed for the items at the subscale level as well as the whole questionnaire.

### Exploratory factor analysis

EFA was performed through the Kaiser-Meyer-Olkin (KMO) test and Bartlett’s test of sphericity. Values above 0.7 confirm the adequacy of the data for conducting EFA [21]. In addition, eigenvalues and scree plot were utilized to determine the number of factors. An eigenvalue is a measure that determines the amount of variance in a dataset explained by a factor; therefore, factors with higher eigenvalues explain more variance [22].

Factor analysis assesses inner-variable relations and is used to extract a group of items that are most closely related to each other. In this analysis, items with factor loadings of < 0.3 were omitted, and the research team decided whether to accept or omit those with factor loadings between 0.3 and 0.5 [22].

### Confirmatory factor analysis

To assess the structure of factors obtained from the exploratory factor analysis, the model was fitted using the confirmatory factor analysis. This factor analysis investigates the confirmation of the exploratory model theoretically and the relationship between factors. The fitness of indices was used to evaluate the model fitness. The following indicators were considered to confirm the acceptable model: Root Mean Square Error of Approximation (RMSEA) < 0.08, Standardized Root Mean Square Error of Approximation (SRMSEA) < 0.08, Comparative Fit Index (CFI) ≥ 0.90, Tucker- Lewis Index (TLI) ≥ 0.95, Normed Chi-square (× 2/ df) < 5.0.

### Reliability

To determine the overall reliability of the questionnaire, internal consistency and test-retest reliability were measured. Internal consistency was assessed by calculating Cronbach’s alpha in a sample of 20 mothers. Based on rule of Thum, the sample size for reliability is about 10–20% of the total sample size [23]. The test-retest reliability was also assessed by calculating ICC for the same participants who completed the questionnaire twice at a two-week interval.

### Ethical consideration

This study was taken from a PhD thesis in midwifery approved by the Ethics Committee of Tabriz University of Medical Sciences (Ethics code: IR.TBZMED.REC.1398.066). Informed written consents were obtained from all the participants.

## Results

A majority of eligible women (96.1%) agreed to participate in the study and 109 pregnant women who did not participate in the childbirth classes were excluded from the study. A total number of 205 mothers were enrolled from September 2019 to January 2020. The mean (SD) age of the participants was 27 (5.2) years, and most of the participants (80.5%) were housewives (Table 1). The language spoken by the researcher and the participants was Azeri but they were fluent in Persian which is the official language of Iran.

**Table 1:** Characteristics of the study participants (*n* = 205)

Characteristics	N (%)
Age (Years)^a^	27.0 (5.2)
Education	
Secondary school or below	31 (15.1)
Diploma and high School	85 (41.5)
University	89 (43.4)
Job	
Housemaker	165 (80.5)
Employee	40 (19.5)
Income	
Not at all sufficient	15 (7.3)
Relatively sufficient	143 (69.8)
Completely sufficient	47 (22.9)
Parity	
Primiparous	98 (47.8)
Multiparous	107(52.2)

^a^Mean (SD)

### Face and content validity

Considering that in Iran, birth preparation classes are taught by trained midwives; hence, the researchers decided to replace “physiotherapist performance” and “anesthesiologist performance” with “midwife performance” in items 7 (training massage) and 8 (pain reduction techniques). All 25 items were described as simple, relevant, and unambiguous. The impact scores were all above 1.5; therefore, the face validity of the instrument was confirmed. The results of CVI and CVR values of each items were acceptable (Table 2). Additionally, the CVI and CVR values for the whole questionnaire were 0.88 and 0.94, respectively.

**Table 2:** The impact score, CVI, and CVR for each questions (*n* = 10 Expert)

Items	Impact score	CVI^a^	CVR^b^
1	4	0.92	1
2	4	0.92	1
3	4	0.92	1
4	4	0.88	1
5	4	0.88	1
6	4	0.80	1
7	3.6	0.80	0.77
8	3.6	0.80	0.77
9	4	0.88	1
10	4	0.92	0.77
11	4	0.88	1
12	3.6	0.84	0.77
13	3.6	0.96	0.77
14	4	0.92	1
15	3.6	0.84	0.77
16	4	0.92	1
17	4	0.92	1
18	4	0.84	1
19	4	0.92	1
20	4	0.92	1
21	4	0.92	1
22	4	0.92	1
23	4	0.92	1
24	4	0.92	1
25	4	0.80	**1**

^a^Content Validity Index, ^b^Content Validity Ratio

### Reliability

Cronbach’s alpha coefficients of the assessed constructs were high: 0.83 for the class structure, 0.92 for the class process and 0.89 for the class outcome).A coefficient of 0.93 was obtained for the whole questionnaire confirming the internal consistency of the questionnaire. Through the test-retest method, an ICC of 0.96 (0.89–0.98) was obtained for the whole questionnaire, and those of the class structure, the class process, and the class outcome were 0.85 (0.62–0.94), 0.98 (0.96–0.99), and 0.93 (0.83–0.97), respectively (at a 95% CI).

### Exploratory factor analysis

**Fig. 1:**
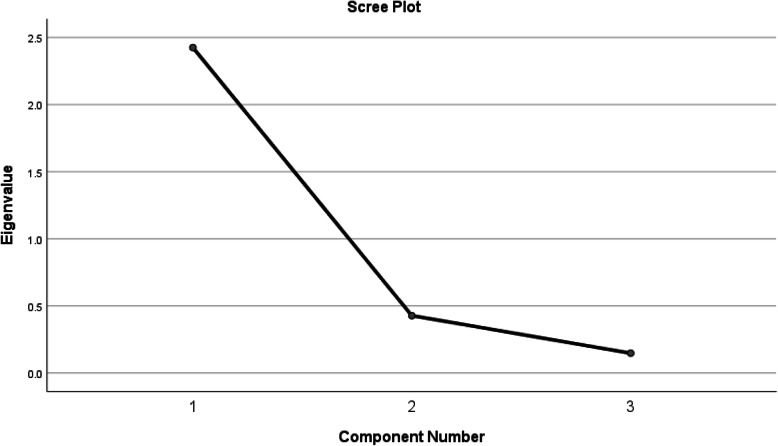
Scree Plot for subscales (exploratory factor analysis for three subscales of the questionnaire)

EFA was performed in two steps. The first step was carried out for each subscale, separately. KMO values for the subscales of the class structure, the class process, and the class outcome were 0.818, 0.921, and 0.794, respectively. The results of Bartlett’s test was statistically significant which demonstrated the data adequacy for performing a scale-based EFA (*P* < 0.001). Based on scree plot, items of each subscale load only on one factor; thus, the convergent construct validity was confirmed for all subscales. The total variance explained values for the three subscales were 71.74, 60.774 and 67.967, respectively. The second step of EFA was performed for all subscales at the questionnaire level, at which KMO value was 0.647, and Bartlett’s *p*-value was smaller than 0.001. Considering a maximum total variance of 80.83, the Iranian version of SCEQ can be predicted by three factors (Fig. 1). The scree plot is used to determine the number of factors to retain in an exploratory factor analysis (EFA). In multivariate statistics, a scree plot is a line plot of the eigenvalues of factors or principal components in an analysis [22].

In addition, since each subscale loads only on one factor (based on scree plot), the convergent construct validity was confirmed for the whole questionnaire. Factor loadings obtained for all items were larger than 0.3; therefore, none of the items were omitted (Table 3). Factor loadings for all the subscales were also above 0.3 (Table 4).

**Table 3:** Factor loadings of the women’s satisfaction with the childbirth education class questionnaire (*n* = 205)

	Factor 1	Factor 2	Factor 3
**Structure of the class**			
Date of the class	.906		
Time of the class	.900		
Length of the class	.914		
Physical environment of the classroom	.792		
Size of the class	.704		
**Process of the class**			
Performance of the midwife		.785	
Performance of the midwife in training massage		.819	
Performance of the midwife in training pain reduction techniques		.830	
Participation in the class		.759	
Amount of information given		.835	
Usefulness of the topic: labour process		.781	
Usefulness of the topic: introduction to labour ward		.734	
Usefulness of the topic: husband’s role		.740	
Usefulness of the topic: preparation for the labour		.866	
Usefulness of the topic: breathing exercise and relaxation technique		.891	
Usefulness of the topic: pain relief in labour		.816	
Effectiveness of teaching method: didactic teaching		.892	
Effectiveness of teaching method: demonstration		.784	
Effectiveness of teaching method: practice		.744	
Effectiveness of teaching method: audiovisual materials		.656	
Effectiveness of teaching method: tour to labour ward		. 428	
**Outcome of the class**			
Ability to fulfil your informational need			.799
Ability to give you courage for labour			.857
Ability to reduce your anxiety for labour			.892
Overall impression of the class			.742

Extraction Method: Principal Axis Factoring

**Table 4:** Factor loadings of the subscales of SCECQ

subscales of questionnaire	Factor loadings
Structure of the class	0.880
Process of the class	0.952
Outcome of the class	0.862

The overall validity of the model was confirmed due to the results in CFA with the X^2^/df ratio above 5, and RMSEA below 0.08 (Table 5). Moreover, all goodness-of-fit indices (including GFI, AGFI, NFI, NNFI, RFI, IFI, and CFI) were greater than 0.9, indicating that the research model fits the data well. The correlations between all factors were significant (*P* < 0.001) (Table 6).

**Table 5:** Confirmatory factor analyses fit Index of the women’s satisfaction with the childbirth education class (*n* = 205)

Fit Indices (DFS)	Fit
χ2	981.563
df	266
P	< 0.001
$$ \raisebox{1ex}{${x}^2$}\!\left/ \!\raisebox{-1ex}{$ df$}\right. $$	3.690
GFI	0.932
AGFI	0. 972
NF1	0. 933
RF1	0. 912
IF1	0. 972
NNF1	0. 955
CF1	0. 972
RMSEA (90%CI)	0.070 (0.065; 0.076)

**Table 6:** Significant Correlations between subscales

			Correlations Estimate	S.E	C.R	P
Satis_1	<−->	Satis_3	.787	.034	7.980	> 0.001
Satis_1	<−->	Satis_2	.720	.037	7.579	> 0.001
Satis_3	<−->	Satis_2	.910	.031	8.288	> 0.001

**Fig. 2:**
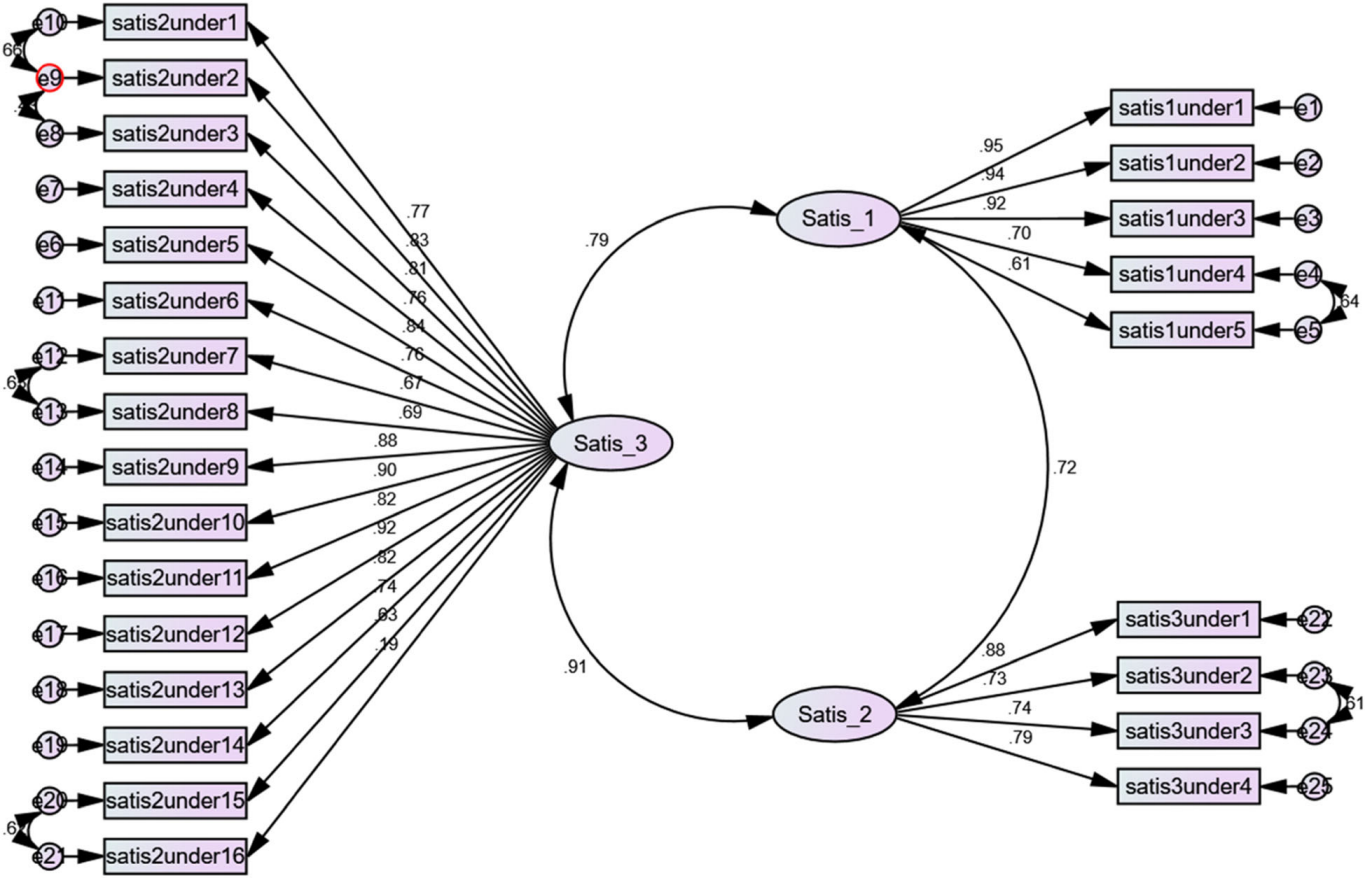
CFA factor loading- Coefficients of item-scale relationship for 25 items of questionnaire (3 Ovals represent subscales of SCECQ and rectangles represent items of subscales, numbers next to the arrows indicate correlation coefficients)

Figure 2 illustrates a path diagram with standardized coefficients representing the conceptual research model. The minimum and maximum coefficients of item-scale relationship were 0.61 and 0.95 in 5 items of structure subscale, 0.73 and 0.88 in 4 items of outcome subscale, 0.19 and 0.92 in 16 items of process subscale of questionnaire. All coefficients of subscales and items relationship in the confirmatory factor analysis were significant (*p*-value < 0.001 for all subscales and ite, p-value = 0.007 for 16th item of subscale process of class) demonstrating that all items were significantly correlated with their factor.

## Discussion

This study assessed the psychometric properties of SCECQ for Iranian pregnant women. According to the results, the Persian version of this questionnaire is a reliable and valid instrument for assessing Iranian pregnant women’s satisfaction with childbirth education classes. In the psychometric assessment of the Persian version of the questionnaire using factor analysis, three factors of structure, process, and outcome from the original questionnaire were confirmed to predict the satisfaction of women from their childbirth education classes. Factor analysis is a commonly used technique for assessing construct validity [24].

The KMO was used as a measure of data adequacy for EFA [25]. KMO values for the subscales of class structure, class process, and class outcome were 0.818 (suitable), 0.921 (very suitable), and 0.794 (acceptable) respectively. The KMO value for the whole questionnaire was acceptable (0.647).

The minimum factor loading required to accept an item in a subscale depends on the number of items, scales, and eigenvalues. Factor loadings between 0.3 and 0.4 were considered acceptable [26], and those ≥0.6 were considered very high [27]. In this study, the calculated factor loadings ranged from 0.42 to 0.89, an interval indicating high validity of the items.

CFA was performed to assess the construct validity of the instrument. In this respect, it is necessary to have a good understanding of appropriate events, which are independent assessment indices [28]. The chi-square (X^2)^ test results indicate whether there is a significant difference between the implied and the observed covariance matrices [29]. In this study, the observed matrix was compatible (*P* < 0.05). X^2^/df ratios lower than 3 indicate good compatibility, and those lower than 5 indicate moderate compatibility. A ratio of 3.69 was obtained in this study, a finding which indicates moderate compatibility. The obtained goodness-of-fit indices including GFI, AGFI, NFI, NNFI, RFI, IFI, and CFI were > 0.9, a finding which indicates that the research model fits the data well [30].

The resultant Cronbach’s alpha coefficients for the constructs of class structure (0.83), class process (0.92), and class outcome (0.89) and the coefficient calculated for the whole questionnaire (0.93) confirmed the internal consistency of the instrument. These findings are in line with the results of the original questionnaire (alpha = 0.89) [12]. Finally, an overall ICC of 0.93 confirmed the questionnaire.

Measuring satisfaction levels is a very common component of many evaluations of health care quality [31]. Client satisfaction is related to better physical and mental health outcomes but dissatisfaction can lead to unnecessary medical interactions, prevent sharing of information and impair trust-building [32, 33]. The results of this study indicate that the instrument can be used to assess women’s satisfaction with antenatal classes in Iran. Future studies using this instrument may subsidy the planning and conduction of childbirth preparation classes. We also recommend further studies assessing differences between primiparous and multiparous in evaluating their satisfaction with childbirth educational classes.

### Strengths and limitations

This was the first study conducted in Iran to assess the psychometric properties of SCECQ. Due to random sampling of participant and wide range of demographic factors such as different age groups, job and parity, the results of this study can be generalized for Iranian women including primiparous and multiparous. However, there extra studies can take place among women with different cultures who live in rural areas of Iran. The other limitation of the study is about performing EFA and CFA for the same data. Although the external validity of the study could have been increased if the researchers used a larger sample size and divided the participants into two sub-samples to estimate stable parameters [34], due to limitation of time for a PhD study, minimum sample size of participants was used in this study. All the steps in the study was performed based on the protocol except including multiparous women into the study. The reason for that was the possibility of using the questionnaire with primiparous and multiparous women who would take childbirth classes across the country.

## Conclusion

The results showed that the Persian version of SCECQ was a valid and reliable instrument for measuring pregnant women’s satisfaction with childbirth education classes. This instrument can help health authorities and caregivers measure pregnant women’s satisfaction with the childbirth classes and make necessary interventions to provide further support for pregnant women in those classes.

## Supplementary Information

**Additional file 1.** English version of satisfaction with the childbirth education class questionnaire.

**Additional file 2.** Persian version of satisfaction with the childbirth education class questionnaire.

## Data Availability

The datasets used and analysed during the current study are available from the corresponding author on reasonable request.

## Abbreviations

SCECQ: Satisfaction with Childbirth Education Classes
CVI: Content Validity Index
CVR: Content Validity Ratio
ICC: Intra Correlation Coefficient
EFA: Exploratory Factor Analysis
CFA: Confirmatory Factor Analysis
KMO: Kaiser-Meyer-Olkin
RMSEA: Root Mean Square Error of Approximation
SRMSEA: Standardized Root Mean Square Error of Approximation
