# Supplementary material for: Psychometric properties of satisfaction with the childbirth education class questionnaire for Iranian population
Source: BMC Pregnancy Childbirth. 2020 Nov 5;20:669. doi: 10.1186/s12884-020-03349-1 (PMC7643332; doi:10.1186/s12884-020-03349-1)
Supplement: Supplementary file 1 — Additional file 1. English version of satisfaction with the childbirth education class questionnaire. [file 12884_2020_3349_MOESM1_ESM.docx]

**Supplementary file 1:** English version of satisfaction with the childbirth education class questionnaire

**Structure of the class**

1. Date of the class

not at all satisfied □ not satisfied □ no idea □ satisfied □ very satisfied □

1. Time of the class

not at all satisfied □ not satisfied □ no idea □ satisfied □ very satisfied □

1. Length of the class

not at all satisfied □ not satisfied □ no idea □ satisfied □ very satisfied □

1. Physical environment of the classroom

not at all satisfied □ not satisfied □ no idea □ satisfied □ very satisfied □

1. Size of the class

not at all satisfied □ not satisfied □ no idea □ satisfied □ very satisfied □

**Process of the class**

1. Performance of the midwife

not at all satisfied □ not satisfied □ no idea □ satisfied □ very satisfied □

1. Performance of the midwife in training massage

not at all satisfied □ not satisfied □ no idea □ satisfied □ very satisfied □

1. Performance of the midwife in training pain reduction techniques

not at all satisfied □ not satisfied □ no idea □ satisfied □ very satisfied □

1. Participation in the class

not at all satisfied □ not satisfied □ no idea □ satisfied □ very satisfied □

1. Amount of information given

not at all satisfied □ not satisfied □ no idea □ satisfied □ very satisfied □

1. Usefulness of the topic: labour process

not at all satisfied □ not satisfied □ no idea □ satisfied □ very satisfied □

1. Usefulness of the topic: introduction to labour ward

not at all satisfied □ not satisfied □ no idea □ satisfied □ very satisfied □

1. Usefulness of the topic: husband’s role

not at all satisfied □ not satisfied □ no idea □ satisfied □ very satisfied □

1. Usefulness of the topic: preparation for the labour

not at all satisfied □ not satisfied □ no idea □ satisfied □ very satisfied □

1. Usefulness of the topic: breathing exercise and relaxation technique

not at all satisfied □ not satisfied □ no idea □ satisfied □ very satisfied □

1. Usefulness of the topic: pain relief in labour

not at all satisfied □ not satisfied □ no idea □ satisfied □ very satisfied □

1. Effectiveness of teaching method: didactic teaching

not at all satisfied □ not satisfied □ no idea □ satisfied □ very satisfied □

1. Effectiveness of teaching method: demonstration

not at all satisfied □ not satisfied □ no idea □ satisfied □ very satisfied □

1. Effectiveness of teaching method: practice

not at all satisfied □ not satisfied □ no idea □ satisfied □ very satisfied □

1. Effectiveness of teaching method: audiovisual materials

not at all satisfied □ not satisfied □ no idea □ satisfied □ very satisfied □

1. Effectiveness of teaching method: tour to labour ward

not at all satisfied □ not satisfied □ no idea □ satisfied □ very satisfied □

**Outcome of the class**

1. Ability to fulﬁl your informational need

not at all satisfied □ not satisfied □ no idea □ satisfied □ very satisfied □

1. Ability to give you courage for labour

not at all satisfied □ not satisfied □ no idea □ satisfied □ very satisfied □

1. Ability to reduce your anxiety for labour

not at all satisfied □ not satisfied □ no idea □ satisfied □ very satisfied □

1. Overall impression of the class

not at all satisfied □ not satisfied □ no idea □ satisfied □ very satisfied □
