# Supplementary material for: Psychometric properties of satisfaction with the childbirth education class questionnaire for Iranian population
Source: BMC Pregnancy Childbirth. 2020 Nov 5;20:669. doi: 10.1186/s12884-020-03349-1 (PMC7643332; doi:10.1186/s12884-020-03349-1)
Supplement: Supplementary file 2 — Additional file 2. Persian version of satisfaction with the childbirth education class questionnaire. [file 12884_2020_3349_MOESM2_ESM.docx]

**Supplementary file 2:** Persian version of satisfaction with the childbirth education class questionnaire

**ساختار کلاس**

1. روز تشکیل کلاس­ها

اصلاً راضی نیستم 🞏 راضی نیستم🞏 نظری ندارم 🞏 راضی هستم🞏 کاملاً راضی هستم🞏

2. زمان تشکیل کلاس­ها

اصلاً راضی نیستم 🞏 راضی نیستم🞏 نظری ندارم 🞏 راضی هستم🞏 کاملاً راضی هستم🞏

3. طول مدت کلاس

اصلاً راضی نیستم 🞏 راضی نیستم🞏 نظری ندارم 🞏 راضی هستم🞏 کاملاً راضی هستم🞏

4. محیط فیزیکی کلاس درس ( روشنایی، سر و صدا و ...)

اصلاً راضی نیستم 🞏 راضی نیستم🞏 نظری ندارم 🞏 راضی هستم🞏 کاملاً راضی هستم🞏

5. اندازه کلاس

اصلاً راضی نیستم 🞏 راضی نیستم🞏 نظری ندارم 🞏 راضی هستم🞏 کاملاً راضی هستم🞏

**فرایند کلاس**

6. عملکرد کلی ماما

اصلاً راضی نیستم 🞏 راضی نیستم🞏 نظری ندارم 🞏 راضی هستم🞏 کاملاً راضی هستم🞏

7. عملکرد فرد آموزش دهنده ورزش­های دوران بارداری، زایمان و بعد از زایمان

اصلاً راضی نیستم 🞏 راضی نیستم🞏 نظری ندارم 🞏 راضی هستم🞏 کاملاً راضی هستم🞏

8. عملکرد فرد آموزش دهنده روشهای تسکین درد

اصلاً راضی نیستم 🞏 راضی نیستم🞏 نظری ندارم 🞏 راضی هستم🞏 کاملاً راضی هستم🞏

9. مشارکت در کلاس

اصلاً راضی نیستم 🞏 راضی نیستم🞏 نظری ندارم 🞏 راضی هستم🞏 کاملاً راضی هستم🞏

10. میزان اطلاعات داده شده

اصلاً راضی نیستم 🞏 راضی نیستم🞏 نظری ندارم 🞏 راضی هستم🞏 کاملاً راضی هستم🞏

11. سودمندی موضوع آموزش داده شده در مورد روند زایمان

اصلاً راضی نیستم 🞏 راضی نیستم🞏 نظری ندارم 🞏 راضی هستم🞏 کاملاً راضی هستم🞏

12. سودمندی موضوع آموزش داده شده در مورد معرفی بخش زایمان

اصلاً راضی نیستم 🞏 راضی نیستم🞏 نظری ندارم 🞏 راضی هستم🞏 کاملاً راضی هستم🞏

13. سودمندی موضوع آموزش داده شده در مورد نقش شوهر

اصلاً راضی نیستم 🞏 راضی نیستم🞏 نظری ندارم 🞏 راضی هستم🞏 کاملاً راضی هستم🞏

14. سودمندی موضوع آموزش داده شده در مورد آماده سازی برای زایمان

اصلاً راضی نیستم 🞏 راضی نیستم🞏 نظری ندارم 🞏 راضی هستم🞏 کاملاً راضی هستم🞏

15. سودمندی موضوع آموزش داده شده در مورد تمرینات تنفسی و تکنیک آرام سازی

اصلاً راضی نیستم 🞏 راضی نیستم🞏 نظری ندارم 🞏 راضی هستم🞏 کاملاً راضی هستم🞏

16. سودمندی موضوع آموزش داده شده در مورد تسکین درد زایمان

اصلاً راضی نیستم 🞏 راضی نیستم🞏 نظری ندارم 🞏 راضی هستم🞏 کاملاً راضی هستم🞏

17. اثربخشی روش تدریس: تدریس شفاهی

اصلاً راضی نیستم 🞏 راضی نیستم🞏 نظری ندارم 🞏 راضی هستم🞏 کاملاً راضی هستم🞏

18. اثربخشی روش تدریس: نمایش عملی (مثل اصلاح وضعیت، تکنیک­های تنفسی)

اصلاً راضی نیستم 🞏 راضی نیستم🞏 نظری ندارم 🞏 راضی هستم🞏 کاملاً راضی هستم🞏

19. اثربخشی روش تدریس: تمرین­های عملی

اصلاً راضی نیستم 🞏 راضی نیستم🞏 نظری ندارم 🞏 راضی هستم🞏 کاملاً راضی هستم🞏

20. اثربخشی روش تدریس: مواد سمعی و بصری

اصلاً راضی نیستم 🞏 راضی نیستم🞏 نظری ندارم 🞏 راضی هستم🞏 کاملاً راضی هستم🞏

21. اثربخشی روش تدریس: بازدید از بخش زایمان

اصلاً راضی نیستم 🞏 راضی نیستم🞏 نظری ندارم 🞏 راضی هستم🞏 کاملاً راضی هستم🞏

**نتیجه کلاس**

22. توانایی پاسخگویی به نیازهای اطلاعاتی شما

اصلاً راضی نیستم 🞏 راضی نیستم🞏 نظری ندارم 🞏 راضی هستم🞏 کاملاً راضی هستم🞏

23. توانایی دادن شجاعت برای تحمل زایمان

اصلاً راضی نیستم 🞏 راضی نیستم🞏 نظری ندارم 🞏 راضی هستم🞏 کاملاً راضی هستم🞏

24. توانایی کاهش اضطراب شما در مورد زایمان

اصلاً راضی نیستم 🞏 راضی نیستم🞏 نظری ندارم 🞏 راضی هستم🞏 کاملاً راضی هستم🞏

25. تاثیر کلی کلاس

اصلاً راضی نیستم 🞏 راضی نیستم🞏 نظری ندارم 🞏 راضی هستم🞏 کاملاً راضی هستم🞏
